# Temperature-Related Effects of Myocardial Protection Strategies in Swine Hearts after Prolonged Warm Ischemia

**DOI:** 10.3390/antiox11030476

**Published:** 2022-02-28

**Authors:** Anna Maria Tolomeo, Assunta Fabozzo, Ricardo Malvicini, Giada De Lazzari, Paola Bisaccia, Gianluca Gaburro, Diletta Arcidiacono, Denni Notarangelo, Federico Caicci, Fabio Zanella, Massimo Marchesan, Gustavo Yannarelli, Gianfranco Santovito, Maurizio Muraca, Gino Gerosa

**Affiliations:** 1 Department of Cardiac, Thoracic and Vascular Science and Public Health, University of Padova, 35128 Padua, Italy; annamaria.tolomeo@unipd.it (A.M.T.); denni.notarangelo@studenti.unipd.it (D.N.); gino.gerosa@unipd.it (G.G.); 2 L.i.f.e.L.a.b. Program, Consorzio per la Ricerca Sanitaria (CORIS), Veneto Region, 35128 Padua, Italy; ricardomalvi@gmail.com (R.M.); giadadelazzari@gmail.com (G.D.L.); muraca@unipd.it (M.M.); 3 Cardiac Surgery Unit, Hospital University of Padova, 35128 Padua, Italy; fabio.zanella@aopd.veneto.it; 4 Department of Women’s and Children’s Health, University of Padova, 35128 Padua, Italy; paola.bisaccia@studenti.unipd.it; 5 Instituto de Medicina Traslacional, Trasplante y Bioingeniería (IMeTTyB) CONICET—Universidad Favaloro), Buenos Aires 1078, Argentina; gyannarelli@favaloro.edu.ar; 6 Department of Biology, University of Padova, 35128 Padua, Italy; gianluca.gaburro@studenti.unipd.it (G.G.); federico.caicci@unipd.it (F.C.); gianfranco.santovito@unipd.it (G.S.); 7 Gastroenterology Unit, Veneto Institute of Oncology IOV-IRCCS, 35128 Padua, Italy; diletta.arcidiacono@iov.veneto.it; 8 Gomiero srl. Via Gomiero, 18, 35010 Villafranca Padovana, Italy; massimo_marchesan@yahoo.it

**Keywords:** enriched cardioplegia, donation after circulatory death, transplantation, organ reconditioning, oxidative stress

## Abstract

Insufficient supply of cardiac grafts represents a severe obstacle in heart transplantation. Donation after circulatory death (DCD), in addition to conventional donation after brain death, is one promising option to overcome the organ shortage. However, DCD organs undergo an inevitably longer period of unprotected warm ischemia between circulatory arrest and graft procurement. In this scenario, we aim to improve heart preservation after a warm ischemic period of 20 min by testing different settings of myocardial protective strategies. Pig hearts were collected from a slaughterhouse and assigned to one of the five experimental groups: baseline (BL), cold cardioplegia (CC), cold cardioplegia + adenosine (CC-ADN), normothermic cardioplegia (NtC + CC) or normothermic cardioplegia + cold cardioplegia + adenosine (NtC-ADN + CC). After treatment, tissue biopsies were taken to assess mitochondrial morphology, antioxidant enzyme activity, lipid peroxidation and cytokine and chemokine expressions. NtC + CC treatment significantly prevented mitochondria swelling and mitochondrial cristae loss. Moreover, the antioxidant enzyme activity was lower in this group, as was lipid peroxidation, and the pro-inflammatory chemokine GM-CSF was diminished. Finally, we demonstrated that normothermic cardioplegia preserved mitochondria morphology, thus preventing oxidative stress and the subsequent inflammatory response. Therefore, normothermic cardioplegia is a better approach to preserve the heart after a warm ischemia period, with respect to cold cardioplegia, before transplantation.

## 1. Introduction

Although the initial experience of human transplantation, as reported by Barnard et al. [1] in the 1970s, involved using organs derived from donors after circulatory death (DCD), the typical donors’ current profile has shifted toward the brain death type (donation after brain death, DBD) [2], whose main advantage is related to the possibility of maintaining an adequate organ perfusion until their procurement is completed. Nevertheless, due to the increase in life expectancy and the technological advances in the treatment of the end-stage heart failure, the number of patients demanding heart transplant currently exceeds the availability of organs. In this scenario, large efforts have been made by the scientific community to increase the pool of human cardiac donors by the extension of donors’ suitability criteria [3] and re-evaluation of those after circulatory death.

Recently, cardiac DCD has been re-advocated and re-adopted as an alternative source of organs for transplant [4]. The main limitation to widespread use of this alternative “organ reserve” is the delicate management of hearts that inevitably suffer from a certain degree of ischemic insult during the time of donors’ withdrawal of life support (WLS, [4]) and the subsequent stand-off period, which varies based on country laws. During this “unprotected” warm ischemic time (WIT) [5], myocardial cellular damage is established, and it is further exacerbated once the coronary circulation of the heart is restored. The so-called ischemia-reperfusion injury (IRI), indeed, seems to be characterized by the loss of cellular homeostasis due to intracellular disbalances, such as calcium overload and production of reactive oxygen species (ROS), which lead to the activation of apoptotic processes [6]. ROS are commonly produced as a result of cellular metabolism, and, in physiologic concentrations, they are involved in the regulation of growth factors signaling, hypoxic response, inflammation and the immune response in mammalian cells [7]. However, in pathological conditions, the increase in these reactive intermediates cause direct damage to cellular DNA, protein and lipids, in addition to activating pathways of stress response, which initiate a cytokine-mediated cascade that, ultimately, recruits neutrophils and macrophages, amplifying the damage [8]. Several antioxidant enzymes, such as catalase, superoxide dismutase, glutathione peroxidases and peroxiredoxins, represent the first line of defense against the noxious effects of ROS [9,10,11,12]. Catalase (CAT), a 4 Fe-heme enzyme, is involved in the detoxification of hydrogen peroxide, by converting two molecules of hydrogen peroxide into two molecules of water and one of oxygen [13]. Superoxide dismutases (SODs) are a group of metalloproteins, whose catalytic function is the dismutation of superoxide radical into hydrogen peroxide and molecular oxygen. Finally, the glutathione peroxidases (GPxs) are phylogenetically related enzymes, and they belong to a family whose role is scavenging the hydrogen peroxide and lipid hydroperoxides [14,15]. Furthermore, mitochondria are key organelles in a high-energy-consuming organ, such as the heart, and play a central role in the organ’s homeostasis. In the IRI, the mitochondria are the main target of ROS, which leads to the opening of permeability transition pores (PTP), determining mitochondrial swelling, loss of mitochondrial cristae and membrane potential and, eventually, to cell death. In this sense, preserving the integrity and functionality of the mitochondria is essential to assure the organ’s viability. Several clinical and pre-clinical (animal model) strategies of myocardial protection and reconditioning during and after harvest have been proposed. The delivery of therapeutic interventions to resuscitate ischemic hearts and mitigate IRI after DCD have been experimentally [16,17] and clinically [18] applied. The use of hypothermic cardioplegic solution during heart harvesting is performed routinely, but it has been argued that this might not be the optimal solution in DCD, as it may exacerbate the IRI [19,20,21,22]. With this report, we aim to define an innovative protocol able to guarantee appropriate heart protection and to preserve the organ from ischemic damage, before any type of re-perfusion strategy is adopted, with a perspective to also expand the donor pool to DCD organs, which represents a valid solution to the organ shortage emergency. To achieve our purpose, we will evaluate and compare the biochemical and histological changes occurring in hearts harvested after a prolonged (20 min) period of warm unprotected ischemia and then treated with five different types of protection strategies.

## 2. Materials and Methods

### 2.1. Experimental Setting, Tissue Biopsies and Experimental Groups

Hearts were obtained from Italian Duroc pigs slaughtered for human consumption. Hearts’ weight ranged from 350 to 450 g. The protocols followed by the slaughterhouse were consistent with EC regulations 1099/2009 regarding animal health and protection, at the time of slaughter, supervised by the Italian government and approved by the associated legal authorities of animal welfare (Food and Consumer Product Safety Authority). Animals’ death was determined by bleeding after electrical cerebral stunning. Mean time of absolute “warm ischemia” (from animal death to heart on the bench) was 18 ± 3 min (hearts with a longer warm ischemia time were not included in the study). After harvesting, all the surrounding tissues were removed, and the coronary ostia were surgically isolated. Five experimental groups were considered based on the type of treatment administered: (i) baseline (BL) hearts were sampled immediately after harvesting, so they did not undergo any treatment; (ii) 1 L cold cardioplegia (CC, at 4 °C); (iii) 1 L cold cardioplegia + (6 mg/2 mL) adenosine (CC-ADN); (iv) 250 mL of normothermic (room temperature) cardioplegia (NtC) + 750 mL of cold cardioplegia (NtC + CC); (v) 250 mL normothermic cardioplegia supplemented with (6 mg/2 mL) adenosine + 750 mL cold cardioplegia (NtC-ADN + CC). Perfusion time was 15 ± 3 min. Each group was made of three samples to guarantee statistical significance of the experiments; fifteen animals were used for this study, and no animals were excluded from the analysis. The treatments were performed randomly to avoid biases in the surgical procedure. In four groups, the hearts were treated with a crystalloid cardioplegic solution, Celsior^®^ (Genzyme Corp, Boston, MA, USA), chosen as it does not require any addition of blood for preparation, its prompt availability and common usage during heart transplantation (for Celsior^®^ cardioplegic solution composition, see Supplementary Table S1). Additionally, in two groups, adenosine was added to the solution to increase coronary perfusion by reducing vascular resistance—as previously described [23] and currently adopted in several clinical scenarios [24]. The cardioplegic solution was injected directly into the coronary ostia and drained through the venous coronary sinus. After treatment, the hearts were promptly opened, and multiple tissue biopsies (8 mm Ø punches) were taken. All the regions of the heart were sampled: right and left ventricular wall (anterior and posterior), interventricular septum, and right and left atria. Anterior and posterior specimens were not pooled together when being homogenized and were processed separately. Finally, tissue biopsies were analyzed as described below, following the scientific rationale based on cellular mechanisms of response to IRI.

### 2.2. Tissue Samples Preparation

The biopsies derived from the pigs’ hearts were homogenized with Polytron in 4 vol/g of tissue of 10 mM Tris–HCl Buffer pH 7.6, containing 1 mM DTT (dithiothreitol), 0.5 M sucrose and 0.15 M KCl. The homogenates were centrifuged at 20,000 rpm for 60 min at 4 °C (Beckman model J2/21). The supernatants were collected and stored at −80 °C for biochemical analysis, as described below.

### 2.3. Estimation of the Total Amount of Proteins

The total amount of proteins in the cellular extracts was assessed by the Folin phenol reagent method [25], using growing concentrations of bovine serum albumin as standard.

### 2.4. Tissue Cytokines Analysis

Total protein extracts were analyzed to quantify cytokines amount. Pro-inflammatory cytokines and chemokines were simultaneously measured in each protein extract with Luminex xMAP^®®^ technology (Luminex Corporation, Austin, TX, USA). Each measurement was performed in duplicate. Quantitative analyses were performed with Luminex xPONENT 3.1 Software using a five-parameter logistic curve fitting. Data were expressed as pg per mg tissue.

### 2.5. Lipid Peroxidation Assay

The principle of this assay is based on the reaction of a chromogenic reagent, N-metyl-2-phenylindole, with MDA at 45 °C. One molecule of MDA reacts with two molecules of N-metyl-2-phenylindole to yield a stable chromophore with maximal absorbance at 586 nm. The assay was performed according to the kit instruction (Lipid Peroxidation Colorimetric Assay Kit, Oxford Biomedical Research (OBR) Inc., Rochester Hills, MI, USA). Data were normalized on total protein concentration.

### 2.6. Catalase Activity Assay

CAT (EC 1.11.1.6) activity was measured according to Aebi et al. [26], assaying the consumption rate of H_2_O_2_ at 240 nm by the enzyme. For the catalase activity, 1.9 mL of 50 mM KH_2_PO_4_/K_2_HPO_4_ buffer (pH 7.5) was mixed in a cuvette with 100 μL of sample and 100 μL of H_2_O_2_, and, immediately, the absorbance was measured at 240 nm every minute for 5 min. Blanks were obtained, substituting the sample with a homogenization buffer. One unit of activity is defined as the amount of enzyme that catalyzed the dismutation of 1 μmol of H_2_O_2_ per min. Since azide selectively inhibits catalase, it was included in separate runs at a final concentration of 0.33 mM in the assay medium. In this way, from the total consumption of H_2_O_2_, it is possible to separate the effect attributable to catalase from that due to any peroxidases present in the sample. The delta of the absorbance between sample and blank per time unit was taken as the measure of catalase activity. One unit is defined as the quantity of enzyme that catalyzes the dismutation of 1 μmol/H_2_O_2_/min.

### 2.7. Selenium-Glutathione Peroxidase Activity

Enzymatic activity of the Se-GPx (EC 1.11.1.9) was determined by measuring the decrement of NADPH at the spectrophotometer, exploiting the catalytic reaction of glutathione reductase NADPH dependent [27], which specifically utilizes either H_2_O_2_ (Se-dependent activity) as substrate, and assaying the oxidation rate of NADPH at 340 nm by glutathione reductase for the reduction of GSSG. For the activity of Se-dependent GPx, aliquots (0.1 mL) of cell-free extract were mixed with 0.8 mL assay mixture (100 mM potassium phosphate buffer K_2_HPO_4_/KH_2_PO_4_, pH 7.5), containing 1 mM sodium azide (to inhibit the competition of CAT), 0.12 mM NADPH, 1 U/mL of glutathione reductase, 2 mM GSH), 100 μL of cellular extract suitably diluted in homogenization buffer, if necessary, and 100 μL of 20 mM H_2_O_2_ to initiate the reaction. The absorbance value at 340 nm (pick of absorbance of NADPH) was measured every minute for 5 min. Blanks were obtained, replacing the sample with homogenization buffer. Se-independent activity was determined as the difference between the values obtained in the two analyses. The decrease in NADPH concentration was measured at 340 nm for 5 min. One unit of GPx activity equals 1 mol glutathione oxidized per min, that is, the quantity of enzyme able to catalyze the transformation of 1 μL of substrate per minute at 25 °C per mL. Data were normalized against total protein concentration.

### 2.8. SOD Activity Assay

The total SOD (EC1.15.1.1) activity assay was measured according to [28]. The principle of the assay for detecting SOD activity examines the reduction of NBT as detector of ●O_2_ produced through the riboflavin photoreduction, which is inhibited by the SOD (in blanks, there is a total reduction). A SOD unit is defined as the amount needed to induce half-maximal inhibition of this reaction [28].

A solution composed of (for 100 mL) 150 mg of L-methionine, 4 mg of nitro blue tetrazolium (NBT), 4 mL of riboflavin 0.117 mM, 0.4 mL of KCN 20 mM in NaHPO_4_/KH_2_PO_4_ phosphate buffer 50 mM (pH 7.8), was prepared. This solution needs to be maintained in the dark and shaken for 30 min before use. Then, in each glass test tube were put 2.3 mL of the solution and 200 μL of sample, and exposed in a bright chamber for 13 min. The blanks were composed of only 2.5 mL of reactive solution. After 13 min, the absorbance of the samples and blanks was read at 560 nm. The percentage of inhibition of the colorimetric reaction was calculated as follows: % of inhibition = 100 − [(As⁄Ab) * 100], where As = absorbance of the sample, Ab = absorbance of the blank. The percentage of inhibition must be between 40 and 60%; otherwise, the volume of the sample must be changed, maintaining a total volume of reaction of 2.5 mL.

### 2.9. Transmission Electron Microscopy

Small cardiac tissue samples (about 1–2 mm^3^) were fixed in 2% glutaraldehyde plus 2% paraformaldehyde in 0.1 M sodium cacodylate buffer pH 7.4 O.N. at 4 °C, subsequently postfixed in osmium tetroxide 1% in 0.1 M sodium cacodylate buffer for 2 h at 4 °C and embedded in an Epon–Araldite resin mixture. Semithin sections were stained with toluidine blue. Ultrathin sections (60–70 nm) were obtained with a Leica Ultracut EM UC7 ultramicrotome, counterstained with uranyl acetate and lead citrate, and viewed with a Tecnai G2 (FEI) transmission electron microscope operating at 100 kV. Images were captured with a Veleta (Olympus Soft Imaging System, Olympus Italia S.r.l., 20054 Segrate, Italy) digital camera.

### 2.10. Mitochondrial Analysis (%)

Changes in mitochondrial cristae morphology were quantified by counting mitochondria with reduced lamellar density and/or abnormal lamellar structure, and by expressing these mitochondria relative to the total number of mitochondria counted.

### 2.11. Western Blotting

Mitochondria isolation was performed using Mitochondria Isolation Kit (#89874, Thermofisher, Waltham, MA, USA). The pig tissue biopsies were weighted, rinsed, and disrupted in PBS using a pestle in an Eppendorf tube. The samples were centrifuged, and the pellet collected; after cell lyses and two centrifugation rounds, the mitochondria pellet was finally separated by cytosolic fraction, following manufacturer’s instructions, and the samples stored at −20 °C. Mitochondria samples were additionally lysed with a solution of lysis buffer and protease inhibition cocktail (PIC). Total proteins quantification was performed using Pierce BCA Protein Assay Kit (#23227, Thermofisher). Cytosolic and mitochondrial samples were loaded in a microplate and analyzed using spectrophotometer at 562 nm. Samples were prepared with 2-mercaptoethanol and heated in a dry bath at 95 °C for 5 min for complete denaturation. For the electrophoresis, a Mini-PROTEAN Tetra Cell by Bio-Rad kit was used with hand-cast 7,5% polyacrylamide gel and Tris-Glycine running buffer; the run was performed at 150 V. Semi-dry electroblotting was performed using a Power Blotter XL system (Thermofisher) and Trans-Blot Transfer Buffer to transfer proteins into PVDF membranes, both performed by Bio-Rad. Afterward, membranes were saturated using blocking buffer Tropix I-BLOCK by Thermofisher Scientific (1 h incubation), in agitation. Membrane probing was performed with anti-OPA1 (#PA1-16991, Invitrogen) and anti-TOM40 (#E6Q3Z, Cell Signaling Technology) primary antibodies with overnight incubation; then, membranes were rinsed with TBS-Tween solution, probed with HRP-conjugated secondary antibody (#65-6120, Invitrogen) and rinsed again. Protein bands were revealed using SuperSignal West Pico PLUS Chemiluminescent Substrate (#34577, Thermofisher) and collected using iBright imaging system. Protein band intensities were analyzed with GelAnalyzer software.

### 2.12. Statistical Analysis

One-way ANOVA with Kruskal–Wallis test were used for multiple comparisons. Data were analyzed using GraphPad software. Median ± range was used. *P*-values: * *p* < 0.05; ** *p* < 0.01; *** *p* < 0.005; **** *p* < 0.0001.

## 3. Results

### 3.1. Combined Normothermic and Cold Cardioplegia Improves Myocardial Protection against Oxidative Stress

**Figure 1:**
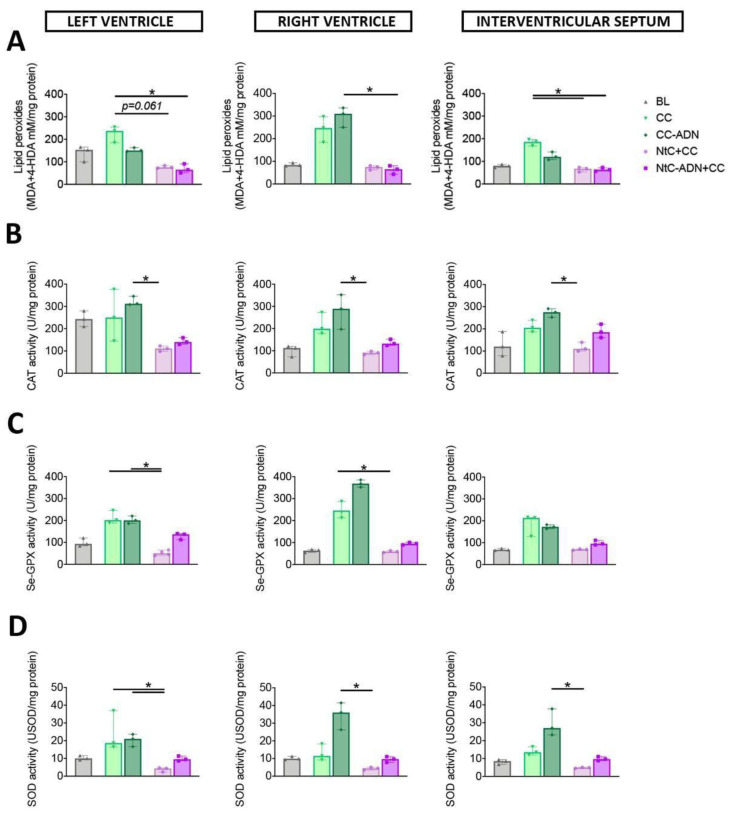
NtC + CC treatment prevents heart ventricle tissue from oxidative damage. Myocardial oxidative stress was quantified as (**A**) Myocardial lipid peroxidation; (**B**) Myocardial catalase activity; (**C**) Myocardial selenium-dependent glutathione peroxidase activity; (**D**) Myocardial SOD cytosolic activity. Values are shown as median ± range, *n* = 3. Note * *p* < 0.05. BL: baseline; CC: cardioplegia; CC-ADN: cardioplegia supplemented with adenosine; NtC + CC: 250 mL warm cardioplegia + 750 mL cold cardioplegia; NtC-ADN + CC: 250 mL warm cardioplegia supplemented with adenosine + 750 mL cold cardioplegia.

**Figure 2:**
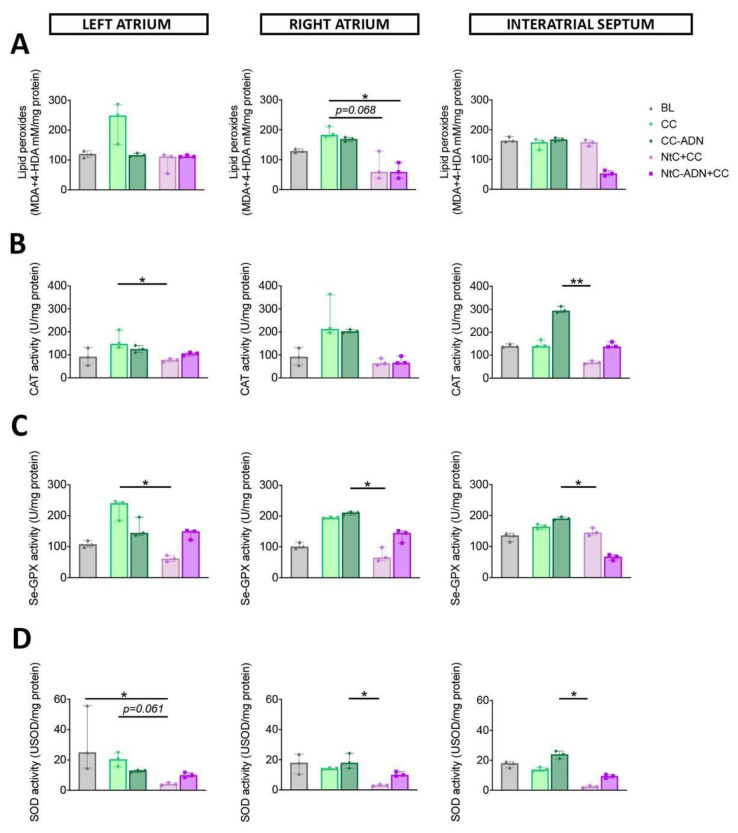
Oxidative damage is prevented when atrial tissue is protected with NtC + CC. Myocardial oxidative stress was quantified as (**A**) Myocardial lipid peroxidation; (**B**) Myocardial catalase activity; (**C**) Myocardial selenium-dependent glutathione peroxidase activity; (**D**) Myocardial SOD cytosolic activity. Values are shown as median ± range, *n* = 3. Note * *p* < 0.05, ** *p* < 0.01. BL: baseline; CC: cardioplegia; CC-ADN: cardioplegia supplemented with adenosine; NtC + CC: 250 mL warm cardioplegia + 750 mL cold cardioplegia; NtC-ADN + CC: 250 mL warm cardioplegia supplemented with adenosine + 750 mL cold cardioplegia.

Lipid peroxidation was used as a marker of oxidative damage. The content of lipid peroxides was significantly lower in the group treated with NtC-ADN + CC, compared to the CC group for the left ventricle (*p* < 0.05), and a trend in lipid peroxidation content was observed for the NtC + CC group (*p* = 0.061). This marker of oxidative stress was also lower for the NtC + CC and NtC-ADN + CC groups for the interventricular septum (*p* < 0.05, for both groups; Figure 1A). Right ventricle showed a significantly lower lipid peroxidation when the tissue was treated with NtC-ADN + CC, compared with CC-ADN (*p* < 0.05; Figure 1A). Furthermore, the enzymatic activity of catalase was significantly lower for the three cardiac regions analyzed in the group treated with NtC + CC compared to the CC-ADN group (*p* < 0.05). Moreover, for the left and right ventricles, the NtC + CC group demonstrated reduced Se-GPX activity than the CC group (*p* < 0.05) and the CC-ADN in the left ventricle (*p* < 0.05; Figure 1C). This myocardial protection was further confirmed by assessing SOD activities, which were significantly lower in the group treated with NtC + CC, compared to the CC-ADN group for all three cardiac regions (*p* < 0.05) and with the CC treated tissues (*p* < 0.05; Figure 1D), suggesting the superiority of the NtC + CC treatment. These results were also observed at the level of the atrial tissues (right atrium, left atrium and interatrial septum) reported in Figure 2.

### 3.2. NtC + CC Treatment Preserves Mitochondrial Morphology and Cristae Structure

**Figure 3:**
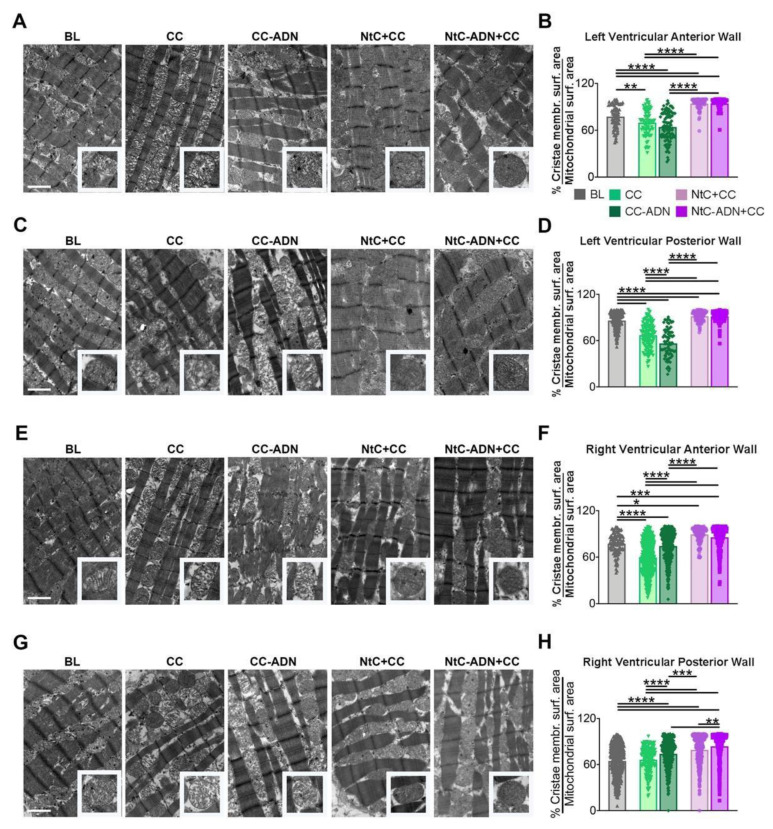
Mitochondrial morphology is preserved by NtC + CC treatment. (**A**–**G**) Transmission electron microscopy (TEM). Several heart regions of left and right ventricular were fixed, cut, and analyzed. Representative images are shown. Bars, 1 μm. Further statistical analyses on mitochondrial cristae are provided below in (**B**,**D**,**F**) and (**H**). A total of 30 images per area were analyzed. Values are shown as median ± range, *n* = 3. Note * *p* < 0.05; ** *p* < 0.01; *** *p* < 0.001; **** *p* < 0.0001. BL: baseline; CC: cardioplegia; CC-ADN: cardioplegia supplemented with adenosine; NtC + CC: 250 mL warm cardioplegia + 750 mL cold cardioplegia; NtC-ADN + CC: 250 mL warm cardioplegia supplemented with adenosine + 750 mL cold cardioplegia.

To further assess the protective effect of the different treatments, we evaluated the tissue ultrastructure and mitochondrial morphology (% cristae membrane surface area/mitochondrial surface area), as damaged mitochondria swell and lose their cristae. When analyzing the left ventricular anterior wall biopsies, we found that NtC + CC and NtC-ADN + CC treatments significantly prevented mitochondrial swelling (as seen from the lower mitochondrial surface area) and prevented the loss of mitochondrial cristae, with respect to mitochondria from hearts treated only with CC or not treated (control hearts) (*p* < 0.0001) (Figure 3A,B). These findings were also confirmed for all the heart regions analyzed herein (left ventricular posterior wall (Figure 3C,D), right ventricular anterior wall (Figure 3E,F) and right ventricular posterior wall (Figure 3G,H). Moreover, there were no differences in mitochondrial integrity between treatments with NtC + CC and NtC-ADN + CC, except for the right ventricular posterior wall, and NtC-ADN + CC proved to be more effective (*p* < 0.01). Taken together, these results suggest that treatments with NtC + CC and NtC-ADN + CC are useful to preserve mitochondrial ultrastructure.

### 3.3. Different Protection Strategies Do Not Affect OPA1 Expression

**Figure 4:**
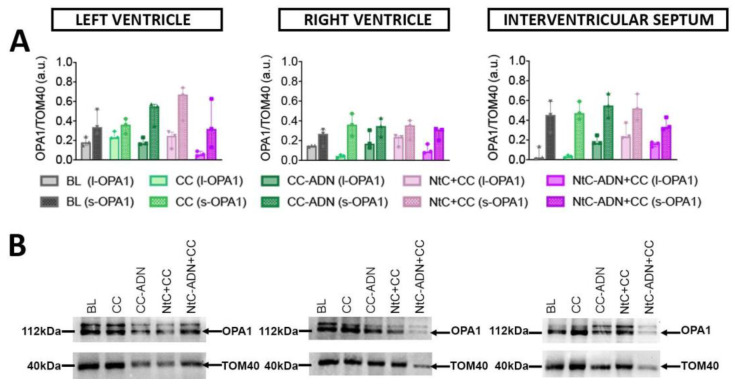
Detection of OPA1 in heart tissue. Quantitative results (**A**) and representative Western blots (**B**) of OPA1 of different heart regions treated with different cardioplegia solutions. TOM40 was used as loading control. Values are shown as median ± range, *n* = 3. BL: baseline; CC: cardioplegia; CC-ADN: cardioplegia supplemented with adenosine; NtC + CC: 250 mL warm cardioplegia + 750 mL cold cardioplegia; NtC-ADN + CC: 250 mL warm cardioplegia supplemented with adenosine + 750 mL cold cardioplegia.

The expression of OPA1, a protein which is a marker of mitochondrial damage, was also investigated. OPA1 is a protein required for mitochondrial fusion and maintenance of normal cristae structure that is deranged during apoptosis. Covalent changes of OPA1 structure caused by oxidative stress might hamper its function, facilitating mitochondrial fragmentation and release of proapoptotic proteins. Specifically, band intensity quantification was performed for both isoforms, l- OPA1 and s-OPA1, for the left ventricle, right ventricle, and interventricular septum (Figure 4A), and for total OPA1 (Supplementary Figure S1). Band intensity was normalized to TOM40 protein. In our study, no significant differences in the expression levels of OPA1 were observed within the treated groups (Figure 4B).

### 3.4. Tissue Cytokines, Chemokines and Tissue Repair Mediators Have a Lower Expression after NtC + CC Treatment

The granulocyte–macrophage colony-stimulating factor (GM-CSF) is a pro-inflammatory cytokine that stimulates stem cells to produce granulocytes and monocytes. We found a significant decrease in the concentration of GM-CSF in the group treated with NtC + CC, with respect to the CC and CC-ADN group, in the left ventricle (*p* < 0.05) and interventricular septum (*p* < 0.05), respectively (Table 1). IL-8 is a chemokine responsible for the recruitment of neutrophils and macrophages to the injury site. We found that treatment with NtC + CC significantly decreased the levels of this cytokine in the left ventricle (*p* < 0.01) and the right ventricle (*p* < 0.05), with respect to the BL and CC treated groups, respectively (Table 1). IL-4, typically produced to counteract an ischemic insult, was lower in the groups where the NtC treatment was administered, with respect to the BL and CC treated groups. Finally, IL-10 was not influenced by the different treatments, but, interestingly, it was undetectable in the LV, while considerable amounts were detected in the RV and IVS.

## 4. Discussion

This report provides an approach toward the development of effective strategies for myocardial protection during organ procurement. Ex-vivo perfusion and implantation become crucial for appropriate clinical translation.

Transplantation still represents the best option when treating patients affected by end-stage organ failure, but the lack of organ donors poses a challenge to adequately face the increasing organ demand. Heart failure is a perfect example, as cardiac transplantation still remains the treatment of choice for end-stage patients. Moreover, heart failure is one of the major causes of morbidity and mortality in Western countries, and approximately 9% of patients on the waiting list for heart transplantation eventually die [29]. Therefore, donor pool expansion [5] is now mandatory, and retrieval of organs from DCD can represent an appealing strategy.

To make DCD transplantation a routine approach, considering the inevitable prolonged no-touch period before the organ can be procured, it is necessary to develop new organ reconditioning strategies to restore organ function immediately after collection. As a matter of fact, extracorporeal reperfusion of explanted organs is already a clinical reality, although it represents a potential source of damage itself (IRI) [2,30], especially in hearts, as it initiates a complex cascade of events that generates a sudden increase in free radicals and tissue inflammatory response [31]. Therefore, an ex-vivo reconditioning strategy, capable of protecting the heart harvested after DCD, and limiting its exposure to ischemic damage and IRI, could lead to a higher availability of organs suitable for transplantation. The current approach for DBD heart preservation relies on administration of hypothermic and hyperkalemic solution at the time of procurement, but the hyperkalemia itself does not have intrinsic cardioprotective properties and can exacerbate IRI [32]. Although this approach is normally used in the case of DBD, where the warm ischemia period is shorter, it is not sufficient to preserve DCD organs.

Moreover, preclinical studies have revealed that normothermic extracorporeal reperfusion can increase organ tolerance to warm ischemia, contributing to preserving organ function and guaranteeing its cellular vitality better than the conventional hypothermic preservation strategy [33,34,35]. In this regard, our study demonstrates that hypothermic myocardial protection is not an appropriate strategy in hearts exposed to prolonged warm ischemia, as hypothermia may itself be a cause of cellular damage.

The production and release of ROS is an injury factor triggered during the hypothermic period in ischemic hearts [36]. These ROS modify DNA, phospholipids, and proteins, leading to DNA adducts, lipid peroxidation and oxidation of thiol groups. These changes play a significant role in the pathogenesis of many diseases, leading to cellular stress and, eventually, to cellular death. Moreover, oxidative stress has been associated with the development and progression of heart failure [37]. In this work, we showed that NtC + CC and NtC-ADN + CC treatments are effective to protect the heart from oxidative damage. Indeed, the NtC + CC and NtC-ADN + CC treatments limited the oxidative insult by significantly reducing the lipid peroxides content in heart tissue. Particularly, according to our results, the NtC + CC perfusion prevented mitochondrial ROS generation. Data were further confirmed by the reduced activity of antioxidant enzymes analyzed in cardiac tissue.

In this scenario, cardiac mitochondria play a dual role in determining cell survival and death following IRI. Mitochondria play an important role in providing energy, adjusting osmotic pressure, calcium balance and pH value, and cell signaling, particularly in the intrinsic pathway of apoptosis. Many stimulators, such as ROS, Ca^2+^ and cytokines, could activate cysteine aspartate-specific proteases (Caspase) by inducing cytochrome C release, which leads to apoptosis [38]. Therefore, preventing mitochondrial dysfunction induced by acute myocardial IRI is an important therapeutic strategy for myocardial protection. In our study, when the tissue ultrastructure and the mitochondrial morphology were evaluated, we found that NtC + CC and NtC-ADN + CC treatments significantly prevented mitochondrial swelling and prevented the loss of mitochondrial cristae, thus preserving mitochondrial integrity.

Moreover, mitochondria are highly dynamic organelles that undergo continuous remodeling through fusion–fission processes. Mitochondrial membrane fission and fusion is essential for generating a dynamic mitochondrial network and regenerative partitioning of damaged components via mitophagy [39]. Membrane rearrangement is essential for organelle function [40,41] and contributes to diversity in mitochondrial membrane shape that can reflect metabolic and physiological specialization [42,43,44]. Stress factors can induce mitochondrial fission production of small organelles that undergo mitophagy. During IRI, mitochondria fission is a characteristic process also led by low ATP levels and ROS production. Furthermore, mitochondria fragmentation is a precondition for mitochondria-stimulated apoptosis processes. Mitochondrial inner-membrane fusion requires OPA1 (Optic atrophy protein 1). Loss of OPA1 function results in a fragmented mitochondrial network, loss of mitochondrial DNA and loss of respiratory function [45,46]. OPA1 is unique for a dynamin family GTPase because it is processed to generate two forms. The unprocessed, N-terminal transmembrane-anchored long form is called l-OPA1. The proteolytically processed short form, which lacks the transmembrane anchor, is called s-OPA1 [47].

In our model, we presume that the short time window was not enough to detect modifications in the expression of OPA1 nor to detect variations regarding the long and short isoforms. To date, key questions still remain unanswered in better understanding the function of different Opa1 conformational states and its role in the context of cardiac ischemia. In this sense, a longer time window should be evaluated to further investigate the role of OPA1 and its forms in the fusion–fission mitochondrial processes.

In addition, exacerbation, and persistence of inflammation, coupled with oxidative stress, have been shown to induce programed cell death and contribute to IRI and organ injury [31]. By assessing the inflammatory response as part of the evaluation of the heart, in our model, we found that in the baseline (BL) group, in the absence of perfusion, pro-inflammatory cytokines (e.g., IL-1α, IL-1β and IL-6) were not detected. This may be due to the fact that the short time window assessed in this work was not enough to induce these cytokines in this group. On the other hand, these pro-inflammatory mediators showed up after perfusion, as detected in the CC group, which may be indicative of a response to tissue damage. Moreover, NtC + CC treatment resulted in significantly lower tissue damage, as seen from the mitochondrial analysis. In this sense, and further supporting these results, the levels of cytokine IL-4 and chemokine IL-8 involved in tissue regeneration, whose release increases upon tissue damage, is significantly lower in the NtC + CC group, with respect to the BL and CC groups. Interestingly, this is also true for the GM-CSF levels, which are significantly lower in the NtC + CC group, with respect to the BL and CC groups. Importantly, in the context of heart transplantation, lower levels of GM-CSF could determine a lower leukocyte infiltration of the tissue, and therefore, a lower inflammatory response. Furthermore, it has been demonstrated that different heart regions (i.e., right ventricle and left ventricle) may have a different expression pattern of inflammatory mediators in response to the ischemic insult [48]. In this sense, even though we did not find any significant difference in the IL-10 levels between treatments, its levels were rather high in the right ventricle and interventricular septum, while IL-10 was undetectable in the left ventricle for all the treatments. The biological interpretation of these data requires further investigation, regarding not only the tissue but also the perfusate, to evaluate the release of cytokines [49].

Moreover, two experimental groups containing adenosine were included in this work. The nucleoside adenosine is a metabolite that elicits coronary vasodilation. Furthermore, it was demonstrated that in hypoxic conditions, under energy depletion, cellular levels of adenosine increase, eliciting an antioxidant activity by inducing antioxidant enzymes activity [50,51]. As a result, adenosine has been extensively investigated for its cardioprotective effects, with controversial results in-vivo. Even though some reports demonstrated that adenosine reduces the inflammatory response [52], attenuates the IRI by downregulation of MAPK38 [53] and reduces the magnitude of high potassium-triggered depolarization [54] in myocardial tissue, others have shown no clear benefit from using it in addition to cardioplegia [55,56]. In our experimental setting, we did not observe any additional benefit of adding adenosine to the warm cardioplegia, as there were no significant differences in the antioxidant enzyme activity among the groups. Further studies should be conducted, as the combination of adenosine with lidocaine and Mg^2+^ (known as ALM cardioplegia) has shown promising results as a fully polarizing cardioplegia in human trials [57].

Finally, normothermic perfusion after circulatory death has been considered and experimentally tested for organs different than hearts (such as liver and lungs) with still controversial results. Despite this, to date, pre-clinical and clinical studies offer only indirect demonstration (function at the time of re-conditioned organ loading) of the tissue integrity before a re-perfusion strategy is adopted [58]. In this scenario, we provided full evidence regarding the biochemical and structural changes that occur in cardiac tissue before adopting any organ reperfusion strategy, demonstrating that normothermic perfusion preserves cardiac tissue significantly better than hypothermic perfusion.

## 5. Conclusions

Normothermic re-perfusion may be the preferable choice when dealing with hearts harvested after prolonged warm “unprotected” ischemia. For instance, cellular structure and mitochondrial integrity are better preserved after initial normothermic reperfusion with a mitigation of the oxidative damage and the IRI’s effects. The implementation of the cardioplegic solution with adenosine seems to reinforce this effect to some extent, but its role seems not to be fundamental. Moreover, our findings may contribute to creating an adequate perfusion protocol after prolonged warm ischemia in DCD hearts, superior to 20 min, as the Italian law mandates. The results we gathered allow us to have a realistic picture of the heart conditions at the time of reperfusion re-start and a comprehensive evaluation of its metabolic status. Additionally, as lactate is shown not to be applicable in DCD hearts, our study may aid to find new measurable markers, such as OPA1 and its subtypes, for a more precise organ metabolic assessment and functionality predictions.

## 6. Limitations

In terms of the limitations of this study, it was performed using hearts coming from a slaughterhouse, therefore the warm ischemic time (around 20 min) could not be precisely calculated, although a realistic approximation was offered, and it was not possible to collect the blood compatible with the organ harvested. This limited the possibility of using a blood cardioplegia and, moreover, the chance of using an ex-vivo perfusion system. Furthermore, sample numerosity was limited to these five groups, as the COVID-19 pandemic dramatically reduced access to the facilities and the resulting availability of organs.

## Figures and Tables

**Table 1:** Cytokines, chemokines and mediators of wound healing and tissue repair.

		BL	CC	CC-ADN	NtC + CC	NtC-ADN + CC
**GM-CSF**	LV	15.7 (3.4)	17.1 (5.4)	15.9 (1.5)	5.6 (5.6) *	9.7 (2.3)
	RV	12.25 (3.3)	15.5 (2.1)	12.9 (2.7)	10.8 (1.6)	8.1 (5.3)
	IVS	15.9 (8.4)	16.2 (2.8)	18.5 (5.1)	7.7 (5.4) *	13.5 (3.8)
**TNF-a**	LV	-	-	-	-	-
	RV	-	-	-	-	-
	IVS	-	-	-	-	-
**INF-g**	LV	-	-	-	-	-
	RV	-	-	-	-	-
	IVS	-	-	-	-	-
**IL-1a**	LV	-	1.5 (0.8)	0.5 (0.8)	1.7 (2.9)	-
	RV	-	-	-	-	-
	IVS	0.5 (0.8)	-	-	-	-
**IL-1b**	LV	3.7 (3.7)	-	7.5 (13.4)	-	-
	RV	-	6.5 (6.5)	2.8 (4.9)	1.5 (2.5)	1.7 (3.1)
	IVS	-	-	-	-	-
**IL-1ra**	LV	15.1 (0.6)	13.7 (1.1)	14.9 (4.1)	13.1 (3.9)	12.7 (5.2)
	RV	13.3 (1.6)	13.6 (3.8)	11.8 (2.7)	10.8 (0.9)	13.7 (3.1)
	IVS	15.4 (1.7)	14.9 (1.4)	16.5 (4.8)	12.6 (2.2)	13.8 (3.7)
**IL-2**	LV	12.3 (2.5)	9.5 (5.5)	13.6 (2.3)	6.4 (4.6)	6.9 (4.2)
	RV	10.3 (1.7)	18.6 (13.4)	11.4 (1.5)	8.2 (4.8)	9.7 (5.9)
	IVS	9.3 (5.5)	12.4 (0.5)	21.3 (11.8)	6.5 (4.6)	9.3 (5.7)
**IL-4**	LV	10.2 (3.3)	6.5 (1.2)	9.1 (5.4)	2.2 (5.4) *	1.9 (2.1) *
	RV	10.5 (2.7)	11.7 (4.3)	8.1 (6.4)	4.3 (5.2) *	0.8 (1.2) *
	IVS	6.1 (7.1)	9.4 (3.2)	10.7 (4.8)	4.8 (8.2)	4.1 (5.1)
**IL-6**	LV	-	-	3.1 (5.1)	-	2 (3.1)
	RV	-	2.5 (2.5)	1.2 (1.4)	1.5 (2.5)	0.8 (1.3)
	IVS	-	-	1.3 (1.9)	1.9 (3.3)	2.1 (3.4)
**IL-8**	LV	28.9 (16.2)	20.4 (10.7)	17.4 (5.2)	13.8 (7.7)	4.8 (4.8) **
	RV	19.7 (6.3)	45.3 (8.9)	35.2 (12.1)	18.1 (0.8) *	30.9 (6.9)
	IVS	28.9 (13.2)	37.6 (5.5)	39.3 (14.4)	21.7 (13.1)	32.6 (19.7)
**IL-10**	LV	-	-	-	-	-
	RV	27.1 (14.1)	40.8 (11.1)	25.7 (13.3)	35.1 (9.4)	28.7 (15.5)
	IVS	35.6 (7.1)	28.9 (4.7)	38.4 (6.1)	47.6 (2.0)	43.4 (6.8)
**IL-12**	LV	-	-	-	-	-
	RV	-	-	-	-	-
	IVS	-	-	-	-	-
**IL-18**	LV	-	-	-	-	-
	RV	-	-	-	-	-
	IVS	-	-	-	-	-

* Values are expressed in pg/mg of tissue and shown as median ± range, *n* = 3. Note * *p* < 0.05; ** *p* < 0.01; BL: baseline; CC: cardioplegia. The symbol (-) means not detected. CC-ADN: cardioplegia supplemented with adenosine; NtC + CC: 250 mL warm cardioplegia + 750 mL cold cardioplegia; NtC-ADN + CC: 250 mL warm cardioplegia supplemented with adenosine + 750 mL cold cardioplegia.

## Data Availability

Data is contained within the article and supplementary material.

## Supplementary Materials

The following supporting information can be downloaded at: https://www.mdpi.com/article/10.3390/antiox11030476/s1, Figure S1: Detection of OPA1 in heart tissue; Table S1: Celsior^®^ cardioplegic solution composition.
