# Supplementary material for: Temperature-Related Effects of Myocardial Protection Strategies in Swine Hearts after Prolonged Warm Ischemia
Source: Antioxidants (Basel). 2022 Feb 28;11(3):476. doi: 10.3390/antiox11030476 (PMC8944743; doi:10.3390/antiox11030476)
Supplement: Supplementary file 1 [file antioxidants-11-00476-s001.zip › antioxidants-1604282-supplementary.pdf]

Supplementary Materials

Table S1. Celsior cardioplegic solution composition [1].

Parameter	Value
Na+ (mmol/l)	100
K+ (mmol/l)	15
Mg2+ (mmol/l)	13
Ca2+ (mmol/l)	0,25
Cl- (mmol/l)	160
Lactobionate (mmol/l)	80
Mannitol (mmol/l)	60
Histidine (mmol/l)	30
Glutamate (mmol/l)	20
Glutathione (mmol/l)	3
pH	7,03
Osmolarity (mOsm/l)	320

Supplementary Figure S1.

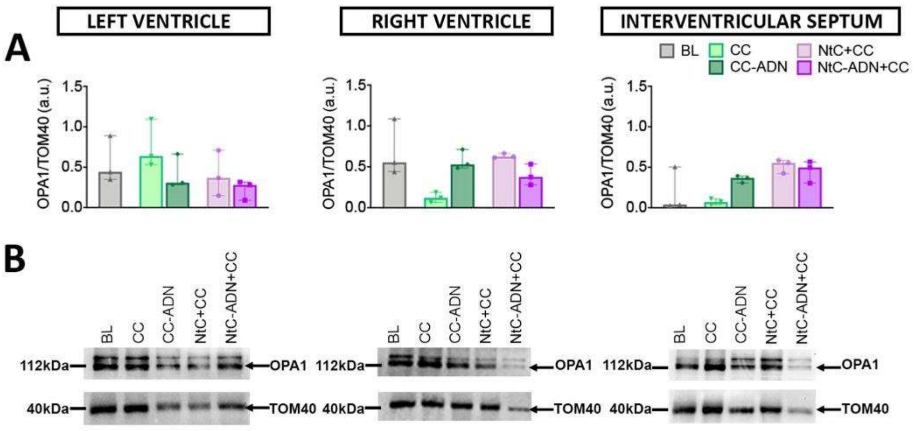

Figure S1. Detection of OPA1 in heart tissue. Quantitative results (A) and representative western blots (B) of OPA1 of different heart regions treated with different cardioplegia solutions. TOM40 was used as loading control. Values are shown as median ± range, n=3. BL: baseline; CC: cardioplegia; CC-ADN: cardioplegia supplemented with adenosine; NtC+CC: 250 mL warm cardioplegia + 750 mL cold cardioplegia; NtC-ADN+CC: 250 mL warm cardioplegia + 750 mL cold cardioplegia supplemented with adenosine.

References

1. Giordano P, Scrascia G, D'Agostino D, Mastro F, Rotunno C, Conte M, Rociola R, Paparella D. Myocardial damage following cardiac surgery: comparison between single-dose Celsior cardioplegic solution and cold blood multi-dose cardioplegia. Perfusion. 2013 Nov;28(6):496-503. doi: 10.1177/0267659113486827. Epub 2013 May 13.
